# The *Bacillus cereus* Food Infection as Multifactorial Process

**DOI:** 10.3390/toxins12110701

**Published:** 2020-11-05

**Authors:** Nadja Jessberger, Richard Dietrich, Per Einar Granum, Erwin Märtlbauer

**Affiliations:** 1 Department of Veterinary Sciences, Faculty of Veterinary Medicine, Ludwig-Maximilians-Universität München, Schönleutnerstr. 8, 85764 Oberschleißheim, Germany; r.dietrich@mh.vetmed.uni-muenchen.de (R.D.); e.maertlbauer@mh.vetmed.uni-muenchen.de (E.M.); 2 Department of Food Safety and Infection Biology, Faculty of Veterinary Medicine, Norwegian University of Life Sciences, P.O. Box 5003 NMBU, 1432 Ås, Norway; per.einar@gmico.no

**Keywords:** *Bacillus cereus*, food poisoning, enterotoxins, outbreaks, spores, motility, adhesion, risk evaluation, toxico-infection

## Abstract

The ubiquitous soil bacterium *Bacillus cereus* presents major challenges to food safety. It is responsible for two types of food poisoning, the emetic form due to food intoxication and the diarrheal form emerging from food infections with enteropathogenic strains, also known as toxico-infections, which are the subject of this review. The diarrheal type of food poisoning emerges after production of enterotoxins by viable bacteria in the human intestine. Basically, the manifestation of the disease is, however, the result of a multifactorial process, including *B. cereus* prevalence and survival in different foods, survival of the stomach passage, spore germination, motility, adhesion, and finally enterotoxin production in the intestine. Moreover, all of these processes are influenced by the consumed foodstuffs as well as the intestinal microbiota which have, therefore, to be considered for a reliable prediction of the hazardous potential of contaminated foods. Current knowledge regarding these single aspects is summarized in this review aiming for risk-oriented diagnostics for enteropathogenic *B. cereus*.

## 1. Introduction

*Bacillus cereus* is a Gram positive, spore-forming and facultative anaerobic rod, which is ubiquitously found in dust, ground, on plant surfaces or in the rhizosphere [1,2,3]. From there, vegetative cells and especially spores can easily enter the food chain via crop plants. According to this, the range of foods in which *B. cereus* is detected is broadly diversified [4,5,6]. Its ability to form spores makes the bacterium highly resistant towards environmental impacts such as drought, heat or radiation, as well as low pH values or chemical conservation [7,8,9]. Thus, it is able to resist the technological processing of foods, which is even enhanced by changed consumers’ demands regarding processed foods [10]. Moreover, the ability of the bacterium to form biofilms complicates cleaning and disinfection measures on surfaces and especially in piping systems of food manufacturing enterprises [11,12,13,14,15].

On the one hand, *B. cereus* plays an important role in foods as a spoilage agent. Its proteolytic and lipolytic properties can cause sensory disorders such as sweet coagulation of milk and cream, the emergence of “bitty cream”, or ropy pastries [16,17,18,19]. On the other hand, the bacterium is best known for its food poisoning abilities. Annual reports of the European Food Safety Authority (EFSA) show that “bacterial toxins other than *Clostridium botulinum*”, including *B. cereus*, generally account for 16–20% of food-poisoning outbreaks, behind Salmonella and viruses. From 2011–2015, 220–291 annual outbreaks associated with *B. cereus* were reported in several member states, which accounted for approximately 3.9–5.5% of all annual food poisoning outbreaks [20,21,22,23,24,25,26]. A current study from France also designates *B. cereus* as one of the most important causes of food poisoning [27]. According to studies performed in the United States, more than one million food-associated illnesses per year are caused by bacterial toxins, including *B. cereus* [28,29,30].

Two types of *B. cereus*-associated gastrointestinal diseases are known, which show a mostly mild and self-limiting course of disease. Nevertheless, severe and fatal outbreaks are also reported [31,32,33,34,35,36,37,38]. The emetic kind of illness manifests in vomiting and nausea, and is caused by cereulide, a small, resistant, ring-shaped dodecadepsipeptide [32,39,40,41,42,43,44,45]. This review focuses on the second form of disease, which is characterized by diarrhea and abdominal pain. The infective dose for this type is estimated from 10^5^–10^8^ cfu/g [46,47] or 10^4^–10^9^ cfu/g [48] (colony forming units per gram of food) vegetative cells or spores. Responsible for the symptoms are different protein enterotoxins, which form pores in the membranes of epithelial cells in the small intestine. These are the tripartite non-hemolytic enterotoxin [49] and hemolysin BL [50], as well as the single protein cytotoxin K [33], which are produced by viable enteropathogenic *B. cereus* in the intestine [48,51]. Predicting the course of disease or evaluating the health risk originating from different enteropathogenic *B. cereus* isolates is difficult, as next to toxin production—which is strain-specifically highly variable itself—a whole range of individual steps has to be considered, which occur during food infection. This review provides an overview on the progress made investigating the single steps of this “multifactorial process”, from prevalence and survival of *B. cereus* in different foods over spore germination, motility, adhesion to epithelial cells and enterotoxin production in the intestine towards a holistic risk evaluation for enteropathogenic *B. cereus*.

## 2. Food Poisoning Outbreaks Associated with *B. cereus*

The bacterium itself was first isolated from an air sample and described by Frankland and Frankland in 1887 [52]. A study from 1906, which was performed after a diarrheal food poisoning outbreak in Germany, initially characterized it as *Bacillus peptonificans* [53]. *B. cereus* was confirmed as the causative organism of gastrointestinal diseases in 1947, when numerous people suffered from diarrhea in Norwegian hospitals after consumption of vanilla sauce [54]. The emetic form of disease was described only 20 years later, when corresponding food poisoning cases appeared in Great Britain after the consumption of cooked rice. It was also postulated for the first time that *B. cereus* produces at least two different types of toxins, which are responsible for either the diarrheal or the emetic type of disease [55,56,57]. Early foodborne outbreaks and clinical manifestations caused by *B. cereus* were summarized by Johnson (1984) [5]. Table 1 gives an overview on further published *B. cereus*-associated food poisoning outbreaks from 1906 until 2019. Initially, the emetic syndrome was largely attributed to Great Britain and Japan, while the diarrheal form occurred rather in Northern Europe or the USA, which was explained by regional-specific consumption of food products [6,58]. Newer available publications reflect a broader distribution of *B. cereus*-associated food poisoning outbreaks. Both forms are reported from North and South America, Canada, Great Britain, North and Central Europe, Australia and Asia (Table 1). Due to large country-specific differences in the surveillance and reporting systems, it is difficult to estimate which syndrome appears more often. Furthermore, many cases remain unrecorded, as (i) people with mild symptoms generally do not seek medical attention, (ii) symptoms are often misdiagnosed as clostridial infections or intoxications with *Staphylococcus aureus* enterotoxins, and (iii) *B. cereus* food poisoning is not a reportable disease. Another obstacle is that many reports do not make clear whether the outbreaks were caused by emetic or enteropathogenic *B. cereus* strains [28,59,60,61,62,63,64,65]. In the course of this review, emetic outbreak reports were found slightly more often, which is in accordance with a recent study from Chai and co-workers, who stated that the diarrheal illness appears “somewhat less common” than the emetic form [66]. Moreover, the diarrheal form mainly appears to manifest “only” in gastrointestinal symptoms such as (watery) diarrhea and abdominal cramps, while the emetic form occasionally results in fatal cases of liver failure (see Table 1). Nevertheless, conclusions about the frequency of occurrence need to be drawn carefully due to the above-mentioned surveillance issues.

## 3. Prevalence and Survival of *B. cereus* in Foods

Due to its ubiquitous nature and the formation of highly adhesive endospores, *B. cereus* is found in a great variety of different foods. Gilbert and Kramer (1986) initially suggested that no type of food with a pH value higher than 4.8 could be excluded [110]. Studies on the prevalence of *B. cereus* in different foods have been conducted from a very early stage, but often without differentiation between enteropathogenic and emetic strains [5,6,110,111,112,113,114,115]. As soon as appropriate detection methods were available, it could be shown that emetic strains are mainly associated with starchy foodstuffs such as rice, pasta and pastries, while enteropathogenic strains are found in all kinds of foods including milk products, vegetables, meat products, sauces, soups, puddings, spices, poultry, and sprouts [6,58,116,117]. This was confirmed by Altayar and Sutherland (2005), who detected only four emetic *B. cereus* isolates out of 271 samples of soils, animal faeces and vegetables and concluded that emetic strains are commonly associated with rice, but rarely with other foodstuffs or environments [118]. Table 2 summarizes more recent reports on the prevalence of emetic and enteropathogenic *B. cereus* strains in different foods. Overall, it is particularly remarkable that strains producing diarrheal enterotoxins are reported much more frequently than emetic isolates. However, it must be pointed out that in several studies the *ces* gene cluster (encoding cereulide synthetase) is not investigated or not mentioned [119,120,121], and that several studies do not distinguish between emetic and enteropathogenic *B. cereus* at all [122,123]. If the emetic toxin genes are investigated, their occurrence is rather rare compared to the enterotoxin genes (Table 2 and [124,125,126,127,128,129]). Other studies challenge the tight association of emetic *B. cereus* with starchy foodstuffs and suggest a rather heterogeneous distribution. Nonetheless, they seem to appear less frequently in meat products, vegetables, lettuce or fruits, and show a higher prevalence in potatoes, rice, mushrooms as well as dairy products (see [130] and Table 2). As *B. cereus* (spores) cannot be completely avoided in foodstuffs of these various origins, the definition of clear cfu limits would be crucial. However, except for dried infant formula, this is not consistently regulated within the European Union, and thus, the EFSA only recommends that cfu levels of 10^3^–10^5^/g should not be exceeded [131]. Thus, a precise understanding of especially the course of food infections with enteropathogenic *B. cereus* is of utmost importance to evaluate their hazardous potential.

The first step for this is profound knowledge about survival and growth of *B. cereus* in the different food matrices, which depend mainly on pH and a_w_ (water activity) values, processing and storage temperatures, oxygen availability, and the presence of microflora, but also on their production of bacteriocins, diacetyl, carbon dioxide, hydrogen peroxide, ethanol, or on further food additives [132,133,134,135,136,137,138,139,140,141,142,143,144,145]. While vegetative *B. cereus* cells can mainly be eliminated by mild heat treatment [146], spores are able to survive high temperatures, such as pasteurization or spray drying of milk [147]. Due to this survival and adjacent outgrowth of the competing microflora, growth of *B. cereus* occurs more often in pasteurized than in raw milk [145,148,149]. It has also been observed that spores from mesophilic strains survive food processing and heat treatment better than spores from psychrotrophic strains [150]. When the seven major phylogenetic groups within the *B. cereus* group according to Guinebretiére et al. [151] were investigated, a high thermal resistance was found for group III (mesophilic) and a comparatively low resistance for psychrotolerant group VI [152,153,154]. Furthermore, a positive correlation between spore heat resistance and growth temperatures of the strains was observed [153]. Heat resistance of the spores is also influenced by the nature and components of the used food matrices, such as free fatty acids [143,155]. Due to changed consumers’ preferences, foods are increasingly exposed to milder preservation treatments such as wet heat for one minute at 95 °C [156]. Germination and outgrowth of the resulting damaged spores depends then again largely on the food matrix. This is also the case for spore production in home-stored foods. Rajkovic and co-workers showed that spore formation highly depends on storage conditions and temperature, which can be strain-specific, but not specific for emetic or diarrheal *B. cereus* [157]. Next to heat treatment, further non-thermal technologies for the elimination/reduction of *B. cereus* spores in foods were established, with the aim of significantly reducing or inactiving spores without affecting the integrity and quality of the food, alone or in combination with mild heat treatment. These are pulsed light treatment [158], electron beam irradiation [159,160], continuous ohmic heating [161,162,163], dielectric barrier discharge plasma [164], acidic electrolyzed oxidizing (EO) water and slightly acidic EO water [140,165] coupled with ultrasonication [166,167], UV treatment [168,169], microwave-combined cold plasma treatment [170] and combined treatment with germinant compounds and superheated steam [171].

Despite every effort, *B. cereus* frequently enters different foodstuffs. A large number of studies showed that growth occurs in a temperature range from approximately 8–50 °C, with the highest cfu being reached at 30–42 °C [123,138,151,172,173,174,175]. In 1998, *Bacillus weihenstephanensis* was described as a psychrotolerant species, which is able to grow at refrigeration temperatures and differs phylogenetically from the mesophilic *B. cereus* [176]. Since then, more and more studies have shown germination and growth (up to 10^8^ cfu/g or ml food) of psychrotolerant, non-*weihenstephanensis* members at low temperatures (4–10 °C) during transport and storage. Thus, a “multiemergence of psychrotolerance in the *B. cereus* group” was postulated [4,132,138,151,153,177,178,179,180,181,182,183]. In this context, it was shown that fatty acids from foods enhance growth of *B. cereus* under cold and anaerobic conditions [184]. On the other hand, some foods and conditions seem not to favour sporulation, germination or growth (refrigerated ricotta salata cheese or tofu [174,175,185]).

Regarding pH values, growth of *B. cereus* is mainly observed within a range of pH 5–7.5 [138,145], and the International Commission for the Microbiological Specifications for Foods (ICMSF) defined a pH value of five as the growth limit for *B. cereus* [186]. Carlin and co-workers determined minimal pH values for growth of different *B. cereus sensu lato* strains of 4.59–4.96 [132] and in older studies growth was observed at even lower values [88,187], which were presumably exceptions. These data correspond to small detection rates and minimal to no germination and growth in yogurt due to pH values lower than five and competing microflora [145,188]. On the other hand, *B. cereus* is able to adapt to lower pH values including organic acids by inducing an acid tolerance response [189,190,191,192,193,194,195,196,197,198]. Additionally, spore survival of alkalization during cocoa production has also been shown [199]. Oxygen availability also plays an important role in temperature- and pH-dependent outgrowth of *B. cereus* [138]. A further, unneglectable factor is the competing microflora. Inactivation of germination and growth of *B. cereus* has been shown in fermented milk or slurries, Brie and Gouda cheese by different lactic acid bacteria—also described as biopreservation—in combination with low a_w_ and Eh values, high salt content, low lactose content, aeration and high acidity [143,145,148,149,179,197,200,201,202,203,204,205,206,207]. Next to food acidification, the microflora contributes to nutrient depletion for the pathogen [208,209]. Furthermore, *B. cereus* spores are able to survive in fermented alcoholic beverages [210] or in dried spices and herbs [211] for several weeks. Complicating the prediction of *B. cereus* survival and growth in different foods, all the above-mentioned processes can be highly strain-specific [4,142,145,151,157,212]. Nevertheless, adaptation to low pH and a_w_ seems to be connected with the phylogeny of the *B. cereus* group [132].

In summary, the foodstuffs which favour *B. cereus* survival, spore germination and outgrowth are those with suitable pH value (approximately 5–7.5), a_w_ value (minimum approximately 0.91–0.95), little or no competing microflora, which are additionally improperly heated or stored. Samapundo and co-workers show clearly the reliance of heating temperature, pH and a_w_ value in different food matrices [179].

## 4. Survival of the Stomach Passage

Many properties guaranteeing *B. cereus* spore survival in heat-treated or acidified foods also benefit their survival during stomach passage, which is the first important step in the infection process. In a study from 1990, the median gastric pH of young and healthy adults was 1.7, the duodenal pH 6.1. After food consumption, the gastric pH increased to 6.7 and decreased gradually to its origin in approximately two hours, while the median duodenal pH was reduced to 5.4 [261]. Several models exist for the gastrointestinal transit of *B. cereus*, such as the use of gastric electrolyte solutions [51,262,263], or simulated gastric fluid with addition of urea, digestive enzymes and mucin [264]. Ceuppens and co-workers developed a five-phase-system mimicking the gastrointestinal passage, including the mouth, stomach with gradual pH decrease and fractional emptying, duodenum with high concentrations of bile and digestive enzymes, followed by dialysis for bile reabsorption, and ileum with competing intestinal microbiota [265,266]. In addition, a rat model was developed [267]. It was generally believed that vegetative *B. cereus* cells are barely able to survive the stomach passage. However, Ceuppens and co-workers found approximately 30% vegetative cell survival after two hours in gastric medium with pH 4 [268] and Wijnands and co-workers developed a model according to which 3–26% of ingested vegetative cells can survive the stomach passage, depending on the strains, their growth phase and the age of the consumer [264]. Nevertheless, spores account for the largest portion of survival. Several studies mentioned in chapter three showed adaptation of *B. cereus* especially towards acidification [191,192,193,194,195,196], which can lead to a higher tolerance of the spores towards gastrointestinal stresses and is described as “cross-protection” [269]. *B. cereus* spores showed resistance to any simulation of the gastric passage from pH 2–5 [270], and in a study from 2004, only at pH values < 1.4 a decrease of spore counts could be detected [51]. It has also been observed that spores from mesophilic isolates survived the simulated gastrointestinal passage better than spores from psychrotrophic strains [271]. In another study, spores of 20 enteropathogenic *B. cereus* strains were able to survive in simulated stomach fluid with pH 2. Although high strain-specific differences appeared in the survival rates, they could not be connected with the toxic potential or the origin (food or outbreak) of the respective isolate [263]. The pH and several further factors influencing the survival of *B. cereus* in the gastrointestinal tract were comprehensively summarized by Berthold-Pluta and co-workers [269]. Pepsin, for instance, which is present in the stomach in concentrations of approximately 0.5–1 g/L, had a notable impact on vegetative *B. cereus*, depending on their growth phase as well as their psychrotolerance [269,272]. *B. cereus* is also able to withstand bile in various concentrations, depending on the strain and on the type of accompanying food [262,271]. In contrast to that, vegetative cells were completely eliminated by bile exposure in other studies, while spores showed higher resistance [268,273,274]. Here, it must not be neglected that the antibacterial activity of bile depends on the pH [269]. Moreover, oxygen availability or—in the case of the gastrointestinal tract—depletion significantly alters survival and growth of *B. cereus* [275,276].

Many of the above-mentioned studies emphasize that survival of *B. cereus* vegetative cells and spores during the gastrointestinal transit depends to a large extent on the accompanying food. The general influence of consumed foodstuffs on food infections with enteropathogenic *B. cereus* is extensively summarized in chapter nine. Another unneglectable factor strongly influencing *B. cereus* survival and outgrowth is the intestinal microbiota, which is discussed in chapter 10.

## 5. Germination of Spores

In many studies mentioned above, the tested survival of *B. cereus* of the gastrointestinal passage was closely interwoven with spore germination, which is another prerequisite for the onset of the diarrheal disease. Nevertheless, the majority of studies investigating *B. cereus* spore germination target spores present in foods, to either trigger germination in foodstuffs to eliminate germinated spores/vegetative cells or to completely avoid germination and outgrowth in foods [10,277,278,279,280,281]. *B. cereus* spores can be triggered by nutrient-rich media, by amino acids such as alanine, cysteine, threonine or glutamine, by the purine ribonucleosides inosine and adenosine, by sugars, by heat treatment, or by their combination [279,282,283,284,285]. Only a small number of publications focus on germination of *B. cereus* spores under (simulated) gastrointestinal conditions. Wijnands and co-workers showed that spores from eight out of 11 enterotoxic *B. cereus* strains—all with comparable germination capacity in BHI medium—were triggered by differentiated CaCo-2 cells, none by HEp-2 cells. Thus, induction of germination seems to be strain- as well as cell line-specific. The germinant, which is present in the supernatant of the CaCo-2 cells, stable towards heat and proteolysis, and most likely bound or degraded by the spores, still needs to be identified [286]. Two years later, it was shown that CaCo-2-induced germination of *B. cereus* spores depends on GerI and GerL germinant receptors. Interestingly, only adhered spores were able to germinate, but spores with disrupted *gerI* or *gerL* operon germinated significantly less [287]. As GerI is necessary for germination activated by purine ribosides or aromatic amino acids, it was speculated that such a small molecule released by the CaCo-2 cells might be the trigger [282,287]. Mucin was also able to strain-specifically trigger germination of *B. cereus* spores, alone or in combination with heat treatment. Moreover, multiple genes involved in sporulation and germination were differentially expressed in *B. cereus* F837/76 upon contact with mucin [288]. In 2019, germination of 20 enteropathogenic and apathogenic *B. cereus* strains was comparatively analysed in CGY full medium, in RPMI 1640 cell culture medium and in cRPMI medium, which was pre-incubated with CaCo-2 cells and filtered. Additionally, response to heat treatment was tested. Germination rates were higher in CGY (10–50%) than in cRPMI medium (2–30%). Generally, high strain-specific differences were observed, some spores responded rather to nutrient availability, some rather to heat treatment, and some rather to the CaCo-2-secreted germinant. Additionally, three-year-old spore preparations also showed strain-specific germination [289]. Great strain-specific differences were also observed in earlier germination studies. These were to some extent connected with the ability of the strains to grow at low temperatures [4]. On the other hand, spores from mesophilic strains were described as better germinating in simulated gastrointestinal fluids than spores from psychrotrophic strains [271]. Highly diverse germination was also observed in a comparative study of 12 *B. cereus* strains [290], and emetic *B. cereus* showed lower average germination than strains from other isolates [291]. Van der Voort and co-workers investigated two enteropathogenic and one emetic *B. cereus*, as well as one *B. weihenstephanensis* strain and found major differences in amino acid-, food- or heat-induced germination. Interestingly, some common, “core” germinant receptors were found, as well as 1–3 individual receptors for each strain, although no distinct connection between receptor profile and germination pattern could be determined [292]. Involvement of different germinant receptor profiles or expression patterns was also suggested for the strain-specific induction of germination by CaCo-2 cells [286]. Additionally, further publications describe the occurrence of core germinant receptors as well as the high diversity of strain-specific receptors, receptor clusters or sub-clusters, although different germination responses could not (yet) be connected with receptor patterns [277,283,293]. Nevertheless, spores lacking receptors are massively affected in their response to different germinants [282,284,287,294]. Despite germinant receptor patterns, temperature, nutrient availability and medium composition during sporulation as well as pH or NaCl concentration play an important role in the germination process [277,290,295,296,297,298].

## 6. Motility and Flagella

After spore germination, the ability to actively move towards their site of action provides a big advantage for enteropathogenic *B. cereus*, especially regarding colonization of the host at the intestinal epithelium [299,300,301]. This has already been shown for other flagellated pathogens such as *Listeria monocytogenes*, *Escherichia coli*, *Helicobacter pylori, Vibrio cholerae* or *Salmonella* spp. [302,303]. *B. cereus* is generally capable of swimming and swarming motility. When swimming of 20 enteropathogenic and apathogenic strains was compared on CGY soft-agar at 30 °C, high strain-specific differences were detected. At 37 °C, diameters of 13 out of 20 strains increased suggesting that motility of *B. cereus* is temperature-dependent to some extent, but primarily highly strain-specific [289]. In this, *B. cereus* clearly differs from invasive pathogens such as *L. monocytogenes*, which loses its ability of active movement due to loss of flagella at higher temperatures [304]. In another study, non-pathogenic strains were generally less motile than food poisoning or clinical strains [305]. It was also found that swimming of different *B. cereus* strains was enhanced in the presence of mucin, and that these strains were able to actively move towards mucin, with swimming radius partially depending on the mucin concentration. This corresponded with a differential expression of genes involved in motility and chemotaxis upon contact with mucin, including flagellar and chemotaxis proteins [288]. Swimming and swarming motility, as well as bacterial pathogenicity, depends strongly on flagella [301,302,306,307,308]. Beyond motility, flagella generally play an important role in adhesion, biofilm formation, colonization or invasion, secretion of effector molecules, tissue penetration, phagocytosis and immune system modulation [302,306]. Flagellin also works as a specific ligand triggering innate immunity [307]. Studies on *B. cereus sensu lato* strains showed a correlation between swarming and hemolysin BL secretion in 42 isolates, with clinical isolates being more motile than food isolates [309]. Moreover, an *flhA* mutant could synthesize, but no longer export flagellin, leading to impaired swimming and swarming motility as well as defective secretion of hemolysin BL and phosphatidylcholine-specific phospholipase C [310]. The production of further virulence-associated factors as well as *plcA* and *hblC* transcription was impaired in the *flhA* mutant [311]. An *fliY* mutant was deficient in chemotaxis and hemolysin BL secretion, leading to the conclusion that FlhF is required for chemotaxis and swarming motility of *B. cereus*. This was confirmed by transcriptional data when 118 genes were differentially expressed during swarming, including flagellar genes and the *hbl* operon [312]. Furthermore, a loss of FlhF resulted in decrease of flagellin, Hbl L2, bacillolysin, sphingomyelinase, PC-PLC, PI-PLC and cytotoxin K, as well as in an increase of NheB, cereolysin O and enolase in the secretome. Pathogenicity against *Galleria mellonella* larvae was also weakened [313]. It was thus stated that swarming is strongly connected with pathogenicity, including the regulation of flagellar arrangement, motility behavior and protein secretion [313,314,315,316]. Interestingly, bile salts reduced motility of *B. cereus* due to down-regulation of motility genes [274]. It has also been shown that flagella, pili and motility play an important role in biofilm formation of *B. cereus* [12,317,318,319] as well as in *B. cereus* endophthalmitis [320,321,322].

## 7. Adhesion to the Intestinal Epithelium

Just as important as active movement towards the intestinal epithelium is the ability of *B. cereus* to stay there. Only pathogens able to dwell in the intestine for a certain time are relevant for the infection. Adhesion to the epithelium guarantees the escape from natural cleaning mechanisms of the intestine, persistence, host colonization and pathogenicity [323,324,325,326,327]. An initial barrier is the intestinal mucus layer, which functions as a lubricant, a carrier for antimicrobial molecules, or a pathogen trap [328,329]. Adhesion to, penetration of and degradation of mucins has been shown for a variety of pathogenic bacteria [324,328,330,331,332]. Probiotic *B. cereus* strains are able to adhere to porcine gastric mucin, with higher adhesion of spores than vegetative cells, with S-layer proteins, flagellin and cell-bound proteases being involved [333]. Moreover, adhesion of pathogenic *B. cereus* to mucin was also shown [334,335]. *B. cereus* is further able to degrade mucin and to use it as a growth substrate [288,336]. Major transcriptional changes in a pathogenic *B. cereus* strain were detected upon contact with mucin, including genes involved in adhesion to and degradation of mucin, such as S-layer proteins, proteases, chitin binding protein, and again flagellin [288].

Next to mucin, adhesion ability to the epithelial cells is equally important for the course of infection. It has been found that *B. cereus* vegetative cells as well as spores are able to adhere to CaCo-2, HeLa and HEp-2 cells, but in a highly strain-specific manner [272,286,289,337,338,339]. The underlying mechanisms are largely unexplored, but an involvement of flagella and especially flagellar component FlhA is verified [339]. Wijnands and co-workers postulated that adhesion of spores occurs unspecific instead of through specific adhesins [286], while another group found evidence that as yet unidentified spore surface molecules specifically bind the protein gC1qR on the surface of human colon carcinoma (CaCo-2) and lung cells [340]. A recent study showed that *B. cereus* virulence is triggered by the interaction of flagellin and the host cell surface-localized glycosphingolipid Gb3 [341]. Surface hydrophobicity seems to be another important factor for adhesion [337], as well as the presence of an S-layer, which can interact with host tissues [338,342]. Furthermore, proteinaceous spore appendages and pili are strain-specifically involved in adhesion [343,344,345]. Evidence has also been found that enterotoxin FM, a cell wall peptidase, is involved in motility as well as adhesion to epithelial cells [346]. Surface hydrophobicity, S-layer proteins, the exosporium, flagella, pili and appendages also play an important role in adhesion of *B. cereus* vegetative cells and spores to inert surfaces [221,343,344,345,347,348,349,350,351].

When 20 enteropathogenic and apathogenic *B. cereus* strains were tested, adhesion of spores to CaCo-2 cells varied strain-specifically between 0.036 and 3%, and adhesion of vegetative cells between 0.45 and 6%, with mean adhesion of vegetative cells higher than that of spores [289]. These rates correspond to earlier studies where the adhesion efficiency of spores was approximately 1% [286]. Nevertheless, there are diverging reports on the adhesion ability of vegetative cells compared to spores [333,337,339], putatively caused by the use of different strains. Interestingly, spores of high toxic strains adhered better to CaCo-2 cells than spores of low toxic strains and spores of food isolates showed higher adhesion than spores of strains isolated from food infections [289]. In other studies, clinical and food poisoning *B. cereus* strains showed significantly higher adhesion to epithelial cells than non-pathogenic strains [305], and periodontal as well as the majority of diarrheal strains adhered to HeLa cells, while this could not be observed for emetic *B. cereus* strains [338]. Auger and co-workers further showed not only adhesion of *B. cereus*, but also of the closely related *B. thuringiensis* to epithelial cells [338].

## 8. Production of Diarrheal Enterotoxins

Once having reached and settled at the intestinal epithelium, the major aspect of *B. cereus*-associated diarrheal food infections is most definitely the production of pore-forming enterotoxins. Production, properties and mechanisms of especially Nhe, Hbl and CytK have been subject of extensive research in recent years. The newly gained knowledge is worth summarizing in a separate review [352]. Briefly, enterotoxin production of *B. cereus* in the host is influenced by a variety of factors, such as the epithelial cells or their secretome, the mucus layer or mucins, sugars, the predominant temperature, pH, oxygen availability, redox conditions, as well as growth phase and sporulation [288,353,354,355,356,357,358,359,360,361,362,363,364,365,366]. Enterotoxin gene expression is a highly complex process depending on the interplay of various transcriptional regulator proteins, which for their part respond to a variety of signals such as carbohydrate and nitrogen availability (CcpA, CodY), energy status of the cell (CodY), oxygen status (ResD, Fnr), phase transition (SinR), or the quorum sensing peptide PapR (PlcR) [355,357,358,366,367,368,369,370,371,372,373]. This complex, concerted interaction might be one explanation for the high strain-specific variability of enterotoxin production in *B. cereus*, combined with strain-specific posttranscriptional or posttranslational modifications, toxin secretion and stability [367,374].

The non-hemolytic enterotoxin (Nhe), which is present in almost 100% of all enteropathogenic *B. cereus* strains, and hemolysin BL (Hbl), present in approximately 50% of these strains [352,375,376,377], consist of three protein components of approximately 35–40 kDa each, with NheA showing sequence homologies to Hbl L2, NheB to Hbl L1 and NheC to Hbl B [378,379,380]. Due to structural similarities to cytolysin A (ClyA), Nhe and Hbl were assigned to the ClyA superfamily of α-helical pore forming toxins [378,381,382,383,384]. The single components of each enterotoxin partly form complexes in solution, but also need a specific binding order as well as concentration ratio at the target cell surface for optimal pore formation and maximum cytotoxicity [385,386,387,388,389,390,391,392,393]. Despite their homology and similarity, pore assembly of Nhe and Hbl differs in some points, such as the occurrence of small, permeable “pro-pores”. Moreover, the single components are not interchangeable [389,391,393]. In contrast to the tripartite enterotoxins, cytotoxin K (CytK) is a single, 34 kDa protein, which belongs to the family of β-barrel pore-forming toxins. Strains expressing the uncommon, but highly toxic variant CytK1 were classified as their own species, *Bacillus cytotoxicus* [33,394,395,396,397,398]. Next to the enterotoxins, which contribute to the largest part of the disease, further (putative) virulence factors such as enterotoxin FM, hemolysins II and III, cereolysin O, phospholipase C, the metalloproteases InhA1 and NprA, further exoproteases, or sphingomyelinase might be involved [289,399,400,401,402,403,404,405,406,407]. It has been shown that the *B. cereus* enterotoxins affect target cells of a great variety of different tissue, origin and species [388,391,408,409,410,411,412,413]. Furthermore, they form pores on planar lipid bilayers [393,414,415], which favored the assumption of rather unspecific cell binding for several years. Nevertheless, just recently, LPS-induced TNF-α factor (LITAF) was determined as the main and its related protein CDIP1 as alternative receptor for Hbl, while a specific binding site for Nhe has still not been discovered [410]. The fate of the affected target cells has also been described as pore formation in the membranes (measured via influx of propidium iodide into the cells) [378,388], cell survival or death (measured via LDH or alkaline phosphatase release and bioassays targeting the respiratory chain) [359,374,409,416], or programmed cell death via apoptotic or inflammatory pathways [408,412,417]. Aside from the enterotoxins, there have been several reports describing *B. cereus* biovar anthracis, a variant harboring the *Bacillus anthracis*-typical plasmid pXO1, which includes genes encoding anthrax-like toxins [418,419,420,421,422,423,424,425,426,427]. This might represent a further, future food poisoning threat.

## 9. Influence of Consumed Foods

Many of the studies mentioned above focused on the sole presence of *B. cereus* vegetative cells or spores in the human gastrointestinal tract, neglecting the fact that ingested bacteria are accompanied by different foodstuffs. Properties and processing of the food might provide valuable information about the course of infection, e.g., is the food acidified, has there been a putative adaptation to low pH values (see chapters three and four), did the spores experience thermal damage, or was germination induced by pre-heating (see chapter five)? Primarily, consumed foodstuffs have a considerable effect on bacterial/spore survival of the stomach passage. Clavel and co-workers mixed gastric electrolyte solution with J broth, half-skim milk, pea soup and chicken. While vegetative cell counts rapidly decreased in gastric electrolyte solution at pH < 4, growth was observed at pH 5 under addition of pea soup. At pH < 1.4, spore counts decreased only under addition of J broth and pea soup, but remained stable under addition of milk and chicken. The authors concluded that spore resistance to gastric acidity depends very much on the consumed food [51]. In a follow-up study they showed that also susceptibility to bile salts strongly depends on the food matrix used, with pea soup allowing growth and Hbl production under the highest bile concentrations tested [262]. Ceuppens and co-workers inoculated *B. cereus* in lasagne verde and found no survival of vegetative cells during simulated gastrointestinal passage despite the presence of potentially protective food components. On the contrary, spores survived [273]. Moreover, mashed potato medium might have stabilized *B. cereus* spores during simulated mouth, stomach and duodenum phase [266]. In another study, spores were highly resistant to gastric medium regardless of the tested food, but survival of vegetative cells was enhanced in the presence of milk or chicken [428]. It has also been shown that survival of *B. cereus* spores of the stomach passage is highly strain-specific [263,271]. Additionally, the effect of milk products on spore survival was highly variable depending on the individual strain. Nevertheless, whereas milk, a follow-on formula and rice pudding barely influenced survival, spores were protected by whipped cream and mascarpone [263]. This might be explained by the products’ high content of proteins and especially lipids. The bacteria are withheld in protein-lipid complexes, which defends them against direct exposure to low pH levels [269]. Thus, survival of *B. cereus* of the stomach passage depends on the one hand on the form of ingested cells, but also to a large extent on the kind and amount of ingested food altering stomach pH and protecting the bacteria from acidity or digestive enzymes. On the contrary, wine lowered the total number of viable *B. cereus* under simulated intestinal conditions by inhibiting the proliferation of vegetative cells after spore germination [429]. The authors concluded that consumption of wine during a meal might reduce the risk of an infection. There are also foodstuffs limiting growth of foodborne bacteria including *B. cereus*, such as cauliflower, broccoli and okara byproducts [430].

Foodstuffs can not only affect *B. cereus* survival in the gastrointestinal tract, but also the enterotoxins as well as their activity against epithelial cells, of which only little information is available to date. On the one hand, enterotoxin production is regulated among other things by nutrient availability (see chapter eight and [361,362,367]), to which the consumed foodstuffs might contribute. Protection of the enterotoxin proteins from digestive enzymes by mucin has been shown [288]. Stabilization of the toxins and protection against heat, acid or enzymatic inactivation might as well apply for foodstuffs, which is supported by an increased heat resistance of Nhe in milk [431]. It was shown that kefir antagonizes the cytopathic effects of *B. cereus* towards CaCo-2 cells by interacting with the eukaryotic cells as well as the bacteria [432,433]. In a recent study, the toxic activity of three *B. cereus* reference strains (*nhe*, *hbl* or *nhe* and *hbl* positive) towards CaCo-2 cells was significantly decreased by milk 1.5%, milk 3.5%, lactose-free milk and a baby follow-on formula. From the individual components, lactoferrin, a skim milk powder and vitamins C, B5 and A showed the highest inhibiting effects. Data further indicated that Hbl might be more affected by the presence of these foodstuffs than Nhe. Tested foodstuffs partially blocked the cell surface towards enterotoxin binding, but rather inhibited the specific interaction (compare chapter eight) of the three single Hbl components [263]. This is supported by more recent observations that enterotoxin components NheB and C bind milk proteins and might thus be hindered in their pore-forming and cytotoxic activity (data not yet published). These findings might explain why—despite frequent isolation of enteropathogenic *B. cereus* from milk and milk products (see also Table 2)—outbreaks of the diarrheal disease associated with these foodstuffs are rare.

## 10. Influence of the Intestinal Microbiota

Next to foods, the intestinal microbiota also strongly influences the faith of *B. cereus* in the intestine. Its composition varies depending on the consumers, their age, or individual dietary habits [434,435]. Nevertheless, existing microbial communities are extremely stable against exogenous bacteria and often impede their growth [436]. Here, the interaction of probiotic and pathogenic bacteria is of special interest [437,438,439,440,441,442]. It has been shown that *Lactobacillus plantarum* inhibits *B. cereus* counts when co-incubated during milk fermentation, as well as adhesion of *B. cereus* to CaCo-2 cells by inhibition, competition, and displacement [443]. Furthermore, *Lactobacillus acidophilus* showed antimicrobial effects towards various pathogens including *B. cereus* [444], and different lactic acid bacteria inhibited germination and outgrowth of *B. cereus* in milk [445,446,447]. *Bacillus amyloliquefaciens* RD7-7 and *Bacillus subtilis* HJ18-4, both isolated from fermented soybean food, significantly reduced growth and toxin production of *B. cereus* [448,449]. Three bacteriocin-producing *B. subtilis* strains isolated from maari showed substrate-dependent antimicrobial activity against *B. cereus* [450]. Metabolites produced by *Lactobacillus johnsonii* CRL1647 and *Enterococcus faecium* SM21 inhibited *B. cereus* vegetative cells and spores in a pH dependent manner [451]. While spores of four different *B. cereus* strains survived and germinated in a simulated gastrointestinal passage (see chapter four), outgrowth of the vegetative cells was hindered by the intestinal microbiota in the final ileum phase. The authors concluded that the composition of the intestinal microbiota is crucial for outgrowth or inhibition of *B. cereus* [266]. Moreover, the intestinal microbiota inhibits not only growth of *B. cereus*, but also its cytotoxic activity. A serine protease secreted by the probiotic *Bacillus clausii* counteracted the cytotoxic effects of *B. cereus* and *C. difficile* toxins on Vero and CaCo-2 cells, as well as hemolysis caused by *B. cereus* toxins [452], and exopolysaccharides produced by lactobacilli and bifidobacteria antagonized the cytotoxic effects of *B. cereus* toxins on CaCo-2 cells as well as hemolysis on erythrocytes [453]. *Lactobacillus delbrueckii* subsp. *lactis* modulated cell response in *B. cereus*-infected epithelial and dendritic cells suggesting positive effects of probiotics on the course of infection [413].

## 11. Risk Evaluation of Foods Contaminated with *B. cereus*

Considering all aspects mentioned in this review, it becomes clear that the course of an infection with enteropathogenic *B. cereus* is hard to predict. On the one hand, there is a high variability of enterotoxin production between different strains, which is determined by complex and dynamic regulatory processes concerning gene transcription, posttranscriptional and posttranslational modifications, as well as toxin secretion and stability, which we are, at the moment, only beginning to understand. The same applies for the presence of further secreted virulence factors and their possible interaction with the enterotoxins. There is further unpredictability owing to the fact that only enterotoxins produced by viable *B. cereus* in the intestine contribute to the diarrheal disease, which means that all aspects mentioned above from prevalence in different foods over survival of the stomach passage, spore germination, motility and adhesion, to toxin production under intestinal conditions, as well as the consumed foodstuffs and the intestinal microbiota must be considered. Moreover, many of the just enumerated facts depend on the individual consumer (age, diet, health, etc.).

Routine food diagnostics contain the microbiological identification of “presumptive *Bacillus cereus*” [454], which means it is initially not differentiated between the members of the *B. cereus* group, to which not only *B. cereus sensu stricto* belongs, but also *B. anthracis*, *B. thuringiensis*, *B. weihenstephanensis*, *B. mycoides*, *B. pseudomycoides, B. cytotoxicus, B. toyonensis,* and further just recently described species [151,395,455,456,457,458,459,460]. If further investigations are conducted, mostly the ability to produce enterotoxins is investigated. Three systems for enterotoxin detection in *B. cereus* culture supernatants are commercially available: BCET-RPLA kit (Oxoid, UK; Hbl L2), TECRA-BDE kit (Tecra Interational, Australia; NheA), and Duopath^®^ Cereus Enterotoxins kit (NheB and Hbl L2). These tests certainly give only vague information about the amount of toxins actually produced and can hardly be used to evaluate a strain’s cytotoxicity [376,388]. Far more advanced, but also more elaborate, are cell culture tests, which show the toxic activity of *B. cereus* toxins/supernatants towards target cell lines [359,374,377,388,391,461,462]. Nonetheless, these tests do not reflect the entire course of an infection with enteropathogenic *B. cereus*. Berthold-Pluta and co-workers concluded in their review in 2015 that it is mainly the interaction between *B. cereus* and enterocytes that is necessary for the diarrheal form of food poisoning to evolve [269]. We go even further and state that the interplay of all steps described above is necessary for the manifestation of the disease. In a recent publication, a risk evaluation scheme based on the behavior of 20 enteropathogenic and apathogenic *B. cereus* strains was established regarding stomach survival, germination, motility, adhesion of vegetative cells and spores as well as enterotoxin production and cytotoxicity towards CaCo-2 cells after growth under laboratory and simulated intestinal conditions [289]. Isolates were characterized as potentially highly pathogenic, pathogenic and apathogenic. This complex virulence assessment scheme correlated well with a faster and comparably easy system based on the detection of NheB, sphingomyelinase and exoprotease activity, which serves as basis for the development of reliable rapid tests for routine diagnostics. The new scheme was also verified by using additional strains from two food poisoning outbreaks in Austria [84,289]. This system should further be complemented with information about the corresponding, contaminated food (prevalence and survival of *B. cereus* in the food itself, and its influence on spore survival or toxic activity depending on its composition, see chapters three and nine). The combined information yields a holistic risk evaluation contributing to the prevention of food poisoning outbreaks with sometimes severe consequences (see chapter one). On the other hand, low-risk foods containing verifiably non-pathogenic *B. cereus* will not be destroyed in vain, which will contribute significantly to food security and prevent economic losses of the manufacturers.

## 12. Conclusions

Despite the often mild and self-limiting course of the diarrheal disease, food infections with enteropathogenic *B. cereus* play an important role firstly in the consumers’ health, and secondly in the food industry, which has to take decisions between product release and blocking once a contamination has been determined. As the ubiquitous spores cannot be completely avoided and cfu limits in foodstuffs are not consistently regulated, the decision depends on scientifically substantiated risk assessment. For this, the precise understanding of the course of infection is of utmost importance. Thus, the many single aspects involved in this multifactorial process, which are reviewed here, must be considered.

## Figures and Tables

**Table 1:** Examples of food poisoning outbreaks caused by *B. cereus* worldwide from 1906 until 2019. The summary of reported cases from 1950 (diarrheal) and 1971 (emetic) to 1985 was taken from Kramer and Gilbert [6]. Crucial former events as well as more recent outbreaks are also summarized. When no year of incidence was indicated, the publication year is shown in ().

**Diarrheal**					
**Year**	**Location**		**Consequences**	**Organism**	**Reference**
1950–1985	Hungary (101–200 reported incidents); Finland (51–100 reported incidents); Bulgaria, Canada, Norway, UK, USA, Sowjet Union (6–50 reported incidents); Australia, Brazil, Chile, China, Denmark, Ireland, Germany, India, Italy, Japan, Netherlands, Poland, Rumania, Spain, Sweden, Yugoslavia (1–5 reported incidents)		Mainly diarrhea	*B. cereus*	[6]
**Year**	**Location**	**Food**	**Affected people/consequences**	**Organism**	**Reference**
(1906)	Germany	Meatballs	300 people, diarrhea, stomach cramps	“*B. peptonificans*”	[53]
(1955)	Norway	Vanilla sauce	4 outbreaks, > 400 illnesses, diarrhea, abdominal pain	*B. cereus*	[54]
(1976)	Great Britain	Meat loaf	Diarrhea, strain 4433/73	*B. cereus*	[55]
(1976)	USA	Vegetable sprouts	Nausea, vomiting, cramps, diarrhea	*B. cereus*	[67]
(1979)	USA	Turkey loaf	28 hospital patients, abdominal cramps, watery diarrhea	*B. cereus*	[68]
(1986)	USA	Rice and chicken in hospital cafeteria	160 hospital employees, mainly diarrhea and abdominal cramps, some vomiting	*B. cereus*	[69]
1985	USA	Beef stew	23 illnesses, cramps, diarrhea	*B. cereus*	[70]
1989	USA	Cornish game hens	55 illnesses, mainly diarrhea and cramps	*B. cereus*	[71]
(1993)	USA	Barbecued pork	139 illnesses, diarrhea, fever	*B. cereus*	[72]
1995	Norway	Stew	152 people, diarrhea	*B. cereus*	[49,73]
1998	France	Vegetable puree	44 illnesses, (bloody) diarrhea, three deaths	*B. cytotoxicus*	[33]
1999	Canada	Mayonnaise	Diarrhea	*B. cereus*	[74]
2000	Italy	Cake	173 people, nausea, watery diarrhea	*B. cereus*	[75]
1954–2004	USA, England	28 isolates from food, stool or vomit	Isolates linked to 11x diarrhea, 11x emesis, 6x no information	*B. cereus*	[76]
1991–2005	Canada	Mainly Asian food, followed by raw food	39 outbreaks, 18 enteropathogenic, mainly abdominal cramps and diarrhea	*B. cereus* *B. thuringiensis*	[77]
2006–2008	India	Not specified	42 diarrheal cases in 2 years	*B. cereus*	[78]
2008	Oman	Hospital meal	58 people, mainly diarrhea, some vomiting	*B. cereus*	[79]
2010	Korea	Lunch buffet	Mainly diarrhea and abdominal pain	*B. cereus*	[80]
2013	Australia	Curried prawns, Caesar salad	125 people, diarrhea, abdominal pain	*B. cereus inter alia*	[81]
(2014)	China	Fermented black beans	139 people, nausea, vomiting, diarrhea; 1 diarrheal isolate	*B. cereus*	[82]
2007–2014	France	Mostly starchy food and vegetables	74 outbreaks, often mix of emetic and diarrheal syndrome, abdominal pain	*B. cereus*, *B. cytotoxicus* (100% *nhe*, 40% *hbl*, 5% *cytK1*)	[27]
2001–2013	Australia	Fish balls, mashed potato and gravy, rice	4 outbreaks, 114 cases total, mainly diarrhea	*B. cereus*	[83]
2013	Austria	1. mashed potatoes 2. pancake soup 3. fruit salad, deer ragout, cranberry-pear	3 outbreaks, mainly diarrhea, some vomiting	*B. cereus*	[84]
2003–2013	Southern Brazil	Mainly cereals, sauce	346 patients, mainly diarrhea and cramps, some vomiting	*B. cereus*	[85]
2016	USA	Refried beans	179 illnesses, 1 diarrheal isolate; mostly vomiting, some diarrhea	*B. cereus*	[86]
2018	Australia	Multi-course-dinner (beef)	Diarrhea and vomiting, mostly enteropathogenic *B. cereus* found	*B. cereus*	[87]
**Emetic**					
**Year**	**Location**		**Consequences**	**Organism**	**Reference**
1971–1985	UK (101–200 reported incidents); Netherlands (51–100 reported incidents); Australia, Canada, Ireland, India, Japan, USA (6–50 reported incidents); Belgium, Bulgaria, Chile, China, Denmark, Finland, France, Germany, Hungary, Norway, Singapore, Spain (1–5 reported incidents)		Mainly emesis, nausea	*B. cereus*	[6]
**Year**	**Location**	**Food**	**Affected people/consequences**	**Organism**	**Reference**
1971	Great Britain	Fried rice	13 illnesses, vomiting, nausea	*B. cereus*	[55,56]
1975	Finland	Boiled rice	18 illnesses, nausea, abdominal pain, vomiting	*B. cereus*	[88]
(1976)	Japan	Chinese noodles	Heart and liver degeneration, death	*B. cereus*	[89]
(1981)	USA	Macaroni and cheese	8 illnesses, nausea, abdominal cramps, vomiting	*B. cereus*	[90]
1981	Singapore	Fried rice	Mainly vomiting abdominal cramps, headache, diarrhea	*B. cereus*	[91]
1993	USA	Chicken fried rice	14 acute gastrointestinal illnesses	*B. cereus*	[92]
1991–1994	Japan	Faecal specimens, foods, not specified	5 outbreaks, emesis	*B. cereus*	[93]
(1997)	Switzerland	Spaghetti and pesto	Vomiting, liver failure, death	*B. cereus*	[34]
1998	USA	Contaminated hands/rice	Emesis	*B. cereus*	[94]
(2003)	Greece	No information	Vomiting, abdominal pain, liver abscess, death	*B. cereus*, presumably emetic	[95]
2003	Belgium	Pasta salad	Vomiting, liver failure, death	*B. cereus*	[31]
(2005)	Finland	Pasta and meat dish	Emesis with diarrhea	*B. cereus*	[96]
2006	Germany	1. Rice dish 2. cooked cauliflower	17 children, vomiting, collapse 1 adult, vomiting	*B. cereus*	[97]
1991–2005	Canada	Mainly Asian food, followed by raw food	39 outbreaks, 5 emetic, mainly abdominal cramps and vomiting	*B. cereus*	[77]
(2008)	Switzerland	Pasta	Abdominal pain, emesis, hepatitis, renal and pancreatic insufficiency, liver failure	*B. cereus*	[36]
(2010)	Japan	Fried rice	Gastroenteritis, acute encephalopathy, liver failure	*B. cereus*	[98]
(2010)	Korea	Cooked and fried rice	Emesis	*B. cereus*	[99]
(2010)	Japan	Reheated fried rice	Vomiting, acute encephalopathy, one dead	*B. cereus*	[38]
1954–2004	USA, England	28 isolates from food, stool or vomit	Isolates linked to 11x diarrhea, 11x emesis, 6x no information	*B. cereus*	[76]
2007	Spain	Tuna fish	Emesis	*B. cereus*	[100]
2007	Germany	Rice pudding	43 children, three adults, emesis	*B. cereus*	[101]
2008	Belgium	Spaghetti	Vomiting, watery diarrhea, death	*B. cereus*	[35]
2004–2006	Korea	Not specified	Sporadic food poisoning cases	*B. cereus*	[102]
(2012)	Belgium	Rice	Family outbreak	*B. cereus*	[103]
2008	France	Pasta	Emesis, abdominal pain, liver failure	*B. cereus*	[104]
2012	Italy	Basmati rice	12 illnesses, mostly vomiting, nausea, abdominal pain; diarrhea	*B. cereus*	[105]
2007–2013	Germany	Different foods	Emetic *B. cereus* in 32 samples, vomiting	*B. cereus*	[106]
(2014)	China	Fermented black beans	139 people, nausea, vomiting, diarrhea; 2 emetic isolates	*B. cereus*	[82]
(2015)	Argentina	Chicken	Vomiting and watery diarrhea, “intermediate isolate”	*B. cereus*	[107]
(2015)	Germany	Rice meal	Vomiting, abdominal pain, liver failure	*B. cereus*	[37]
2007–2014	France	Mostly starchy food and vegetables	74 outbreaks, often mix of emetic and diarrheal syndrome, abdominal pain	*B. cereus* (16% *ces*)	[27]
2001–2013	Australia	Fried rice and honey chicken	1 outbreak, vomiting	*B. cereus*	[83]
2012	Great Britain	Pearl haricot beans	Several nurseries, vomiting	*B. cereus*	[108]
2016	USA	Refried beans	179 illnesses, 6 emetic isolates, mostly vomiting, some diarrhea	*B. cereus*	[86]
(2019)	Germany	Buck wheat	Massive vomiting, diarrhea, esophageal perforation, Boerhaave syndrome	*B. cereus*	[109]

**Table 2:** Examples for the prevalence of enteropathogenic and emetic *B. cereus* strains in different foods worldwide from 1997 until 2020. Data are sorted according to their publication year.

**Enteropathogenic**			
**Food**	**Species**	**Location**	**Reference**
Pasteurized milk	*B. cereus*	Netherlands	[213]
Dietary supplements	*B. cereus*	Scotland	[214]
Milk-based infant formulae	*B. cereus*	Scotland	[120]
Milk and meat products	*B. cereus*	Norway	[4]
Chicken meat products	*B. cereus*	USA	[215]
Fish, meat, milk and vegetable products, oils, flavourings, ready-to-eat foods, pastry	*B. cereus* (mainly enteropathogenic)	Netherlands	[216]
Dried milk products	*B. cereus*	Chile	[217]
Fresh and heat-treated milk	*B. cereus, B. thuringiensis, B. weihenstephanensis*	Poland	[218]
Condiments	*B. cereus*	Africa	[219]
Pasteurized full fat milk	*B. cereus, B. thuringiensis, B. mycoides*	China	[220]
Raw rice	*B. cereus, B. thuringiensis*	USA	[221]
Honey	*B. cereus, B. megaterium*		[119]
Different foods from local markets and restaurants	*B. cereus* (mainly enteropathogenic)	Jordan	[222]
Cooked pasta, lasagne, béchamel and bolognaise sauce, fresh minced beef, fresh-cut vegetables, raw basmati rice	*B. cereus*	Belgium	[179]
Fermented African locust bean Benin condiments	*B. cereus*	Africa/Denmark	[223]
Sunsik (ready-to-eat)	*B. cereus* (emetic and enteropathogenic)	Korea	[224]
Ugba (African oil bean seeds)	*B. cereus*	Nigeria	[225]
Ice cream	*B. cereus*	Turkey	[226]
Potato products	*B. cytotoxicus*	Germany	[227]
Vegetables	*B. cereus*	Mexico	[228]
Fermented soybean paste, green tea, rice, vegetables	Mainly *B. cereus* (emetic and enteropathogenic)	Korea	[229]
Ready-to-eat vegetables	*B. cereus*	Korea	[230]
Bread ingredients and bread	*B. cereus*	Italy	[152]
Spices	*B. cereus, B. thuringiensis*	USA	[231]
Infant formulas, ready-to-eat foods	*B. cereus*	Korea	[232]
Fermented soybean products	*B. cereus*	Korea	[233]
Meat products	*B. cereus*	India	[234]
Fermented soybean products	*B. cereus sensu lato*	Korea	[235]
1489 food samples	5.4% enteropathogenic *B. cereus*	Netherlands	[236]
Milk/dairy farms	*B. cereus, B. thuringiensis*	China	[125]
Fermented soybean food	*B. cereus* (enteropathogenic and emetic)	Korea	[237]
Probiotics	*B. cereus, B. thuringiensis*	China	[238]
Pasteurized and UHT milk	*B. cereus, B. thuringiensis*	Brazil	[239]
Dairy products	*B. cereus* (mainly enteropathogenic)	Ghana	[240]
Pasteurized milk	*B. cereus*	Canada	[241]
Beef products	*B. cereus*	Egypt	[242]
Spices from Asia, India, Mexico, powdered infant formulas, fish feed, dietary supplements	*B. cereus*	USA	[243]
Edible insects	*B. cereus, B. cytotoxicus, B. thuringiensis*	Italy	[244]
Pasteurized milk	*B. cereus* (mainly enteropathogenic)	China	[126]
Powdered infant formula (PIF), mashed potato powder	*B. cereus*, 1 *B. cytotoxicus*	Switzerland	[245]
Cooked food, army catering	*B. cereus* (mainly enteropathogenic)	Switzerland	[246]
Raw vegetables	*B. cereus*	Korea	[247]
Raw milk, dairy products	*B. cereus* (mainly enteropathogenic)	Brazil	[248]
Herbs, spices, cereals, pasta, rice, infant formulas, pasteurized milk, cheeses	*B. cereus* (mainly enteropathogenic)	Poland	[124]
Fresh vegetables and salad	*B. cereus*	Germany	[249]
Cereals, spices, vegetables, seafood, dairy and meat products	*B. cereus* (mainly enteropathogenic)	Tunisia	[250]
Flour products	*B. cereus* (mainly enteropathogenic)	Switzerland	[251]
Potato flakes, millet flour, salted potato chips, soups	*B. cytotoxicus*	Belgium/Mali	[252]
Retail fish, ground beef	*Bacillus* (enterotoxin-positive)	Turkey	[253]
Ready-to-eat foods	*B. cereus* (mainly enteropathogenic)	China	[129]
Vegetables	*B. cereus* (mainly enteropathogenic)	China	[128]
Milk powder, Ras-cheese	*B. cereus* (enteropathogenic and emetic)	Egypt	[254]
Artisanal Mexican cheese	*B. cereus* group	Mexico	[255]
Green leave lettuce	*B. cereus*	Korea	[256]
Ready-to-eat foods and powdered milk	*B. cereus* group	Colombia	[127]
Dairy products	*B. cereus* (mainly enteropathogenic)	China	[257]
Meat	*B. cereus*	Iran	[121]
**Emetic**			
**Food**	**Species**	**Location**	**Reference**
Potato skin	*B. cereus* (4 emetic strains)	Scotland	[118]
Fish, meat, milk and vegetable products, oils, flavourings, ready-to-eat foods, pastry	*B. cereus* (8% emetic)	Netherlands	[216]
Pasta, rice, Asian food, milk products, blackcurrant, honey, parsley	Mainly *B. cereus*	Belgium	[258]
(Boiled) rice	*B. cereus* (cereulide; 7.4–12.9% of samples)	Belgium	[103]
Sunsik (ready-to-eat)	*B. cereus* (emetic and enteropathogenic)	Korea	[224]
Potato	Mainly *B. cereus*	Finland	[259]
Fermented soybean paste, green tea, rice, vegetables	Mainly *B. cereus* (emetic and enteropathogenic)	Korea	[229]
Farinaceous foods, vegetables, fruit, cheese and meat products, sauces, soups, salads	*B. cereus* (1% of 4300 food samples emetic)	Germany	[106]
Fermented soybean products	*B. cereus sensu lato* (17% emetic)	Korea	[235]
1489 food samples	0.067% emetic *B. cereus*	Netherlands	[236]
Milk/dairy farms	*B. cereus, B. thuringiensis* (1% emetic)	China	[125]
Fermented soybean food	*B. cereus* (enteropathogenic and emetic)	Korea	[237]
Cooked rice, pasta, infant formula	*B. cereus*	China	[260]
Pasteurized milk	*B. cereus* (5% emetic)	China	[126]
Powdered infant formula (PIF)	*B. cereus*	Switzerland	[245]
Vegetables, army catering	*B. cereus* (1 emetic strain)	Switzerland	[246]
Raw milk, dairy products	*B. cereus* (2 emetic isolates)	Brazil	[248]
Herbs, spices, cereals, pasta, rice, infant formulas, pasteurized milk, cheeses	*B. cereus* (1.7 and 0.9% emetic)	Poland	[124]
Flour products	*B. cereus* (2 emetic isolates)	Switzerland	[251]
Ready-to-eat foods	*B. cereus* (7% emetic)	China	[129]
Vegetables	*B. cereus* (3% emetic)	China	[128]
Milk powder, Ras-cheese	*B. cereus* (enteropathogenic and emetic)	Egypt	[254]
Dairy products	*B. cereus* (11.1% emetic)	China	[257]
